# Publisher Correction: Adaptive modulation of brain hemodynamics across stereotyped running episodes

**DOI:** 10.1038/s41467-020-20571-9

**Published:** 2020-12-21

**Authors:** Antoine Bergel, Elodie Tiran, Thomas Deffieux, Charlie Demené, Mickaël Tanter, Ivan Cohen

**Affiliations:** 1 Sorbonne Université, CNRS, INSERM, Institut de Biologie Paris Seine-Neuroscience, 75005 Paris, France; 2 Physique pour la Médecine Paris, INSERM U1273, ESPCI Paris, CNRS FRE 2031, PSL Université Recherche, Paris, France

**Keywords:** Hippocampus, Neural circuits, Neuro-vascular interactions, Blood flow

Correction to: *Nature Communications* 10.1038/s41467-020-19948-7, published online 3 December 2020.

The original HTML version of this Article was updated shortly after publication because the previous HTML version contained errors in the links to Supplementary Movies 1–6 and the Reporting Summary. These have been corrected in the HTML version of the article.

## Supplementary information

Supplementary Information

Peer Review File

Description of Additional Supplementary Files

Supplementary Movie 1

Supplementary Movie 2

Supplementary Movie 3

Supplementary Movie 4

Supplementary Movie 5

Supplementary Movie 6

Reporting summary

## Source data

Source Data

