# Supplementary material for: Adaptive modulation of brain hemodynamics across stereotyped running episodes
Source: Nat Commun. 2020 Dec 3;11:6193. doi: 10.1038/s41467-020-19948-7 (PMC7713412; doi:10.1038/s41467-020-19948-7)
Supplement: Supplementary file 1 — Supplementary Information [file 41467_2020_19948_MOESM1_ESM.pdf]

Adaptive modulation of brain hemodynamics across stereotyped running episodes

A. Bergel et al.

Supplementary Material

<u>**Supplementary Figures**</u>

Supplementary Figure 1: LFP electrode implantation procedure and site identification

Supplementary Figure 2: Simultaneous anterior/posterior hemodynamic responses to locomotion in freely-running rats (diagonal plane)

Supplementary Figure 3: Comparison of hemodynamic responses with respect to running direction

Supplementary Figure 4: Comparative analysis of seed-based correlations

Supplementary Figure 5: Details of motor cortex and hippocampal LFP recordings during locomotion

Supplementary Figure 6: Single-trial hemodynamic responses of the locomotion network

Supplementary Figure 7: Brain-wide hemodynamic modulation is directly observable in individual responses to single runs.

Supplementary Figure 8: Hemodynamic modulation affects differently the early and late components of single-trial hemodynamic responses.

Supplementary Figure 9: Vascular dynamics during recovery from isoflurane anesthesia

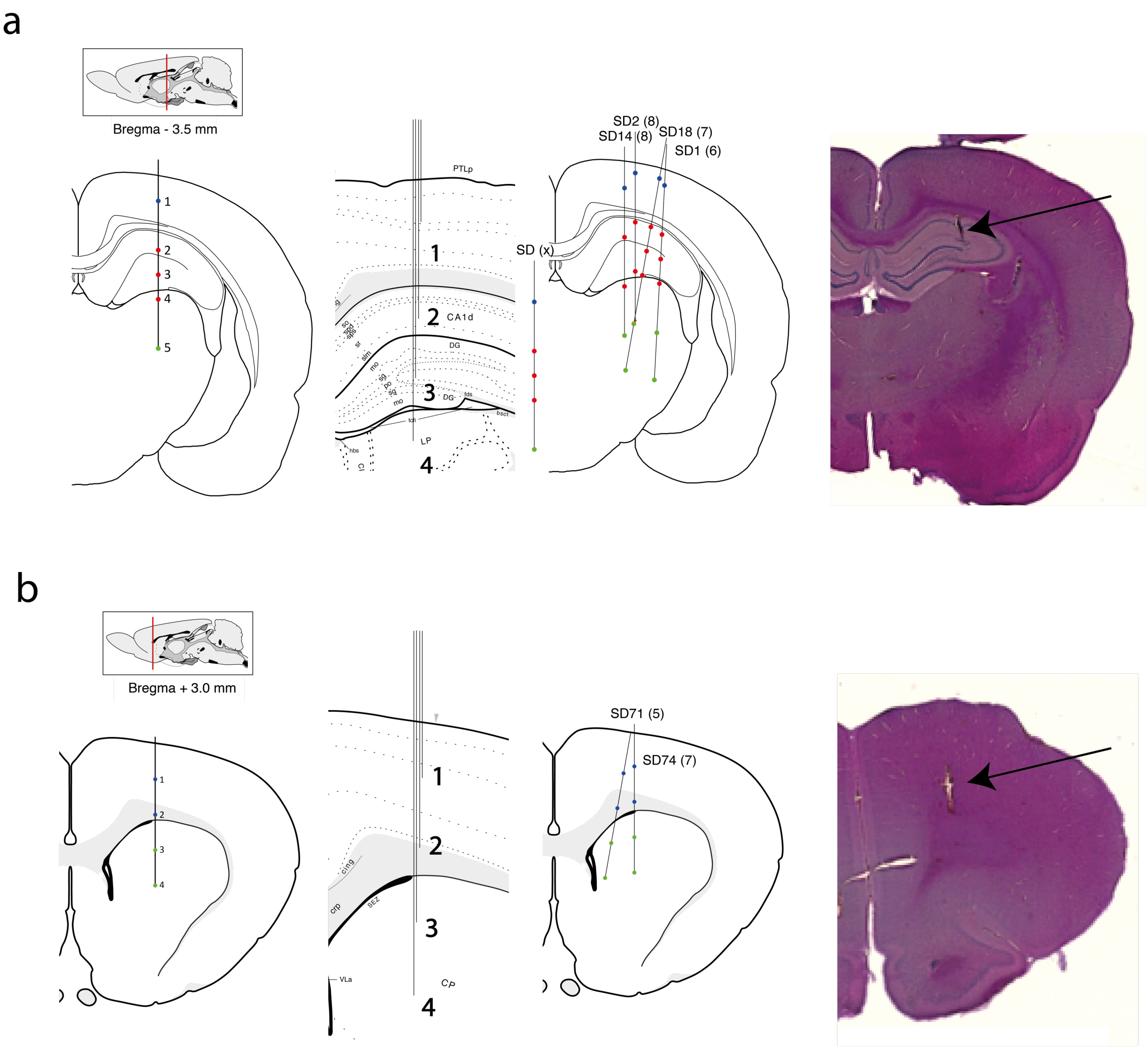

Supplementary Figure 1: LFP implantation procedure and site reconstruction

(a) (Far Left) Schematic representation of targeted recording sites (Coronal plane Bregma – 3.5 mm) (n=7 rats) with one electrode in the cortex (blue), three electrodes in the dorsal hippocampus (red) and one electrode in the dorsal thalamus (green). (Middle Left) Magnified view of the targeted hippocampal recording sites. We aim at recording phase inversion between superficial (site 2) and deep layers (site 3) in the hippocampus. The differential hippocampal LFP signal is the subtraction 2-3, which gives the best signal-to-noise ratio (Middle Right) Post-hoc reconstruction of true electrode location after histological identification for 4 animals (we only displayed animals that were recorded more than 5 times). (Far Right) Typical histological Nissl staining of hippocampal slices showing small lesions at recording sites.

(b) Similar to (a) for motor cortex implantation sites (Coronal plane Bregma + 3.0 mm) (n=2 rats). Targeted recording sites are M1 cortex (blue, sites 1 and 2) and Caudate Putamen (green, sites 3 and 4). The actual design is based on handmade electrodes with minimal spacing of 500 microns between recording sites and a maximal number of 8 electrodes per bundles. Because the surgical procedure is complex, there is variability between targeted structure and actual electrode position due to brain tissue movement (swelling) both during and after the surgery.

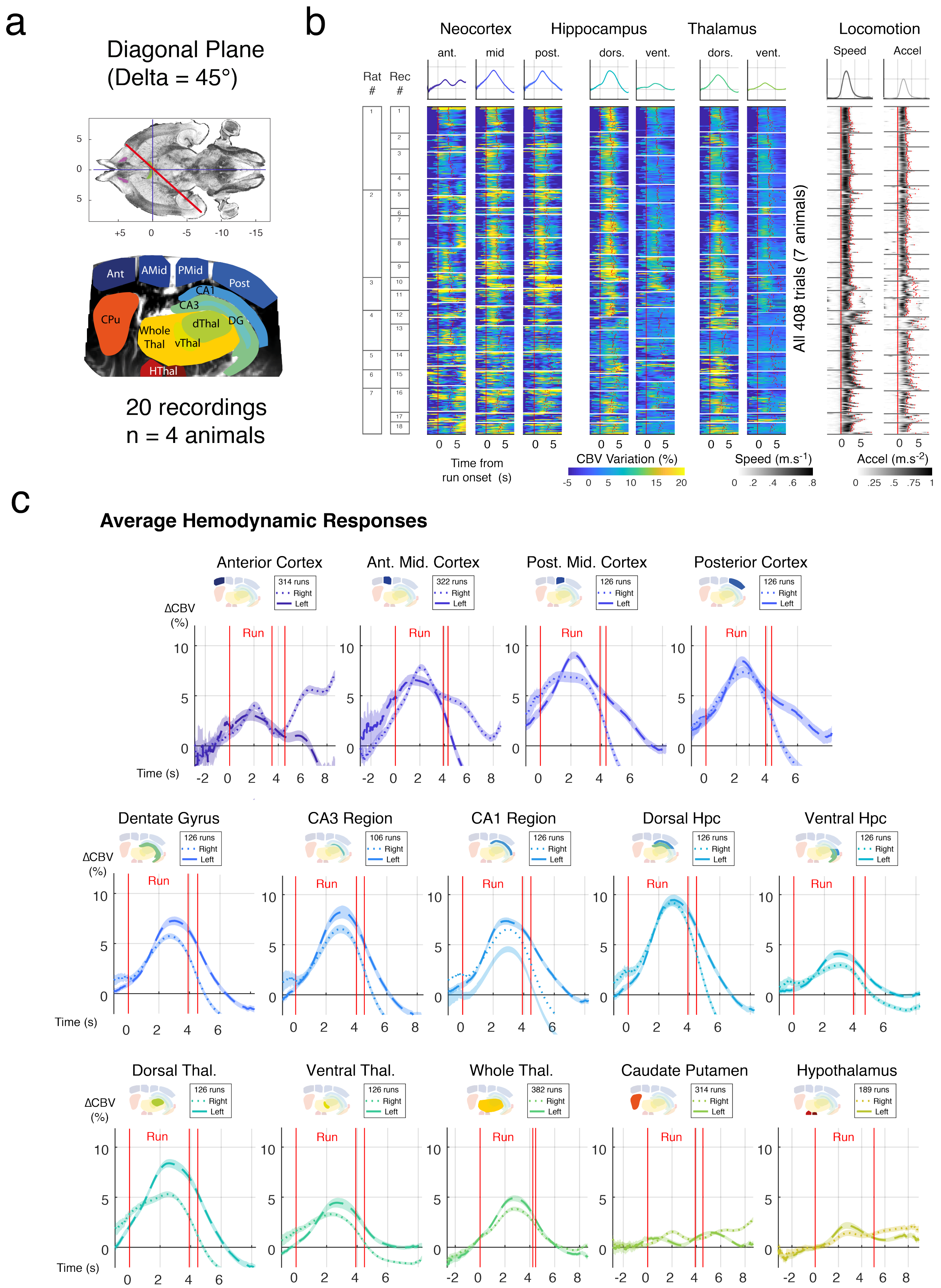

Supplementary Figure 2: Simultaneous anterior/posterior hemodynamic responses to locomotion in freely-running rats (diagonal plane)

(a) Location of a typical diagonal (Delta = 45°) recording plane and associated brain structures monitored during fUS-EEG recordings. Top: Plane position (red) on a transverse view of rat brain

atlas. Bottom Power Doppler image with superimposed atlas registration, performed by positioning salient landmarks onto Power Doppler image and registering a 3D volumetric segmented MRI atlas. Ant: anterior cortex, AMid: anterior midline (parietal) cortex, PMid: posterior midline (parietal) cortex, Post: posterior cortex, DG: dentate gyrus, CA1-CA2-CA3 region, dThal: dorsal Thalamus, vThal: ventral Thalamus, CPu: caudate Putamen, Hthal: Hypothalamus. Reprinted from reference 88, Copyright (2014), with permission from Elsevier.
(b) Bulk representation of single-trial hemodynamic responses to locomotion in seven major brain regions (anterior/midline/posterior Neocortex, ventral/dorsal Hippocampus, ventral/dorsal Thalamus). A total of 408 trials (19 recordings, 7 animals) was acquired across many days. For each run, the onset of movement is used as a temporal reference (zero-timing) and all trials are aligned to run onset (see Methods). Run end however is different for all trials. We can then compute average hemodynamic responses (top) and average run duration (t = 4.26s +/- .3s). The same approach is performed for locomotion parameters: running speed (left) and accelerometer (right). Hemodynamic responses display strong inter-trial variability in the cortical and hippocampal regions, with a strong dissociation between dorsal versus ventral hippocampus and dorsal versus ventral thalamic regions. Horizontal bars delimitate recordings.
(c) Average hemodynamics responses over multiple sub-regions. Using the approach in b, we computed average responses to locomotion in 26 regions. Overall, strong bilateral activations are found in the cortex and all dorsal hippocampus sub-regions (stronger and earlier in the dentate gyrus), together with dorsal thalamus. We display hemodynamic responses for large regions as a reference on the right side. For each region the number of runs can differ slightly as all 26 regions were not always visible on each recording. Error bands correspond to the mean values +/- sem.
Source data are provided as a Source Data file.

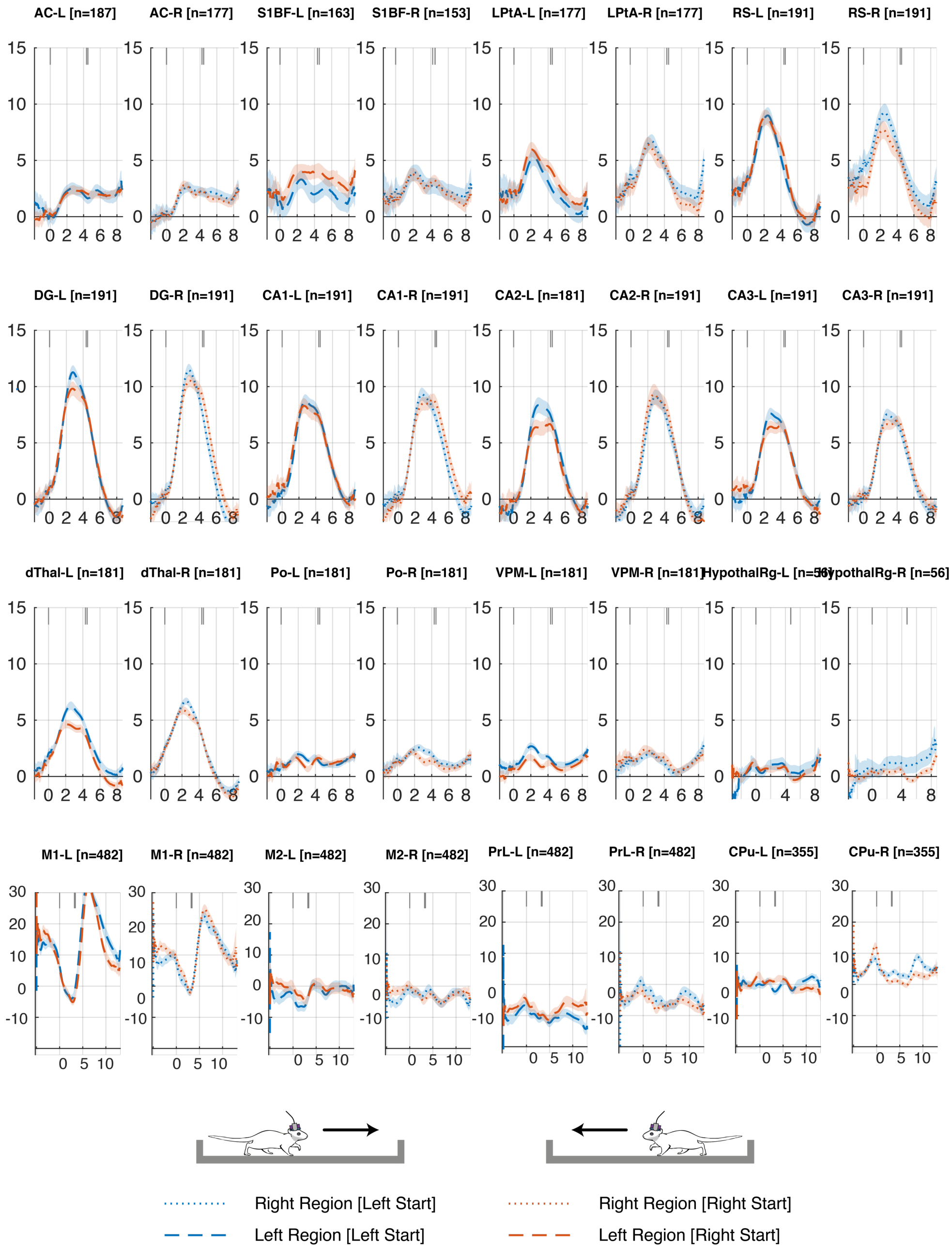

Supplementary Figure 3: Comparison of hemodynamic responses with respect to running direction

Average hemodynamic responses for all coronal recordings segregated according each running direction (n= 7 animals, 19 recordings, 191 left runs, 191 right runs for a total of 388 runs). For each region, the average hemodynamic response for left runs is displayed in blue while the hemodynamic response to right runs is displayed in orange. Brain hemodynamics display very little dependence on running direction both in terms of delay, duration and amplitude. Non-statistically significant differences are visible in the peak amplitude of left dentate gyrus, left CA2 region, left CA3 region and left dorsal thalamus but they were largely influenced by one recording where responses were unilateral. This did not appear on other recordings. More prominent differences are observed within cortical regions (right retrosplenial, left somatosensory, left parietal associative) which might be due to asymmetries during running and reward uptake as some animals showed a preferred rotation side and ran closer to one wall compared to the other[1]. Error bands correspond to the mean values +/- sem. Source data are provided as a Source Data file.

[1] LaMendola, N. P. & Bever, T. G. Peripheral and cerebral asymmetries in the rat. *Science* **278**, 483–486 (1997).

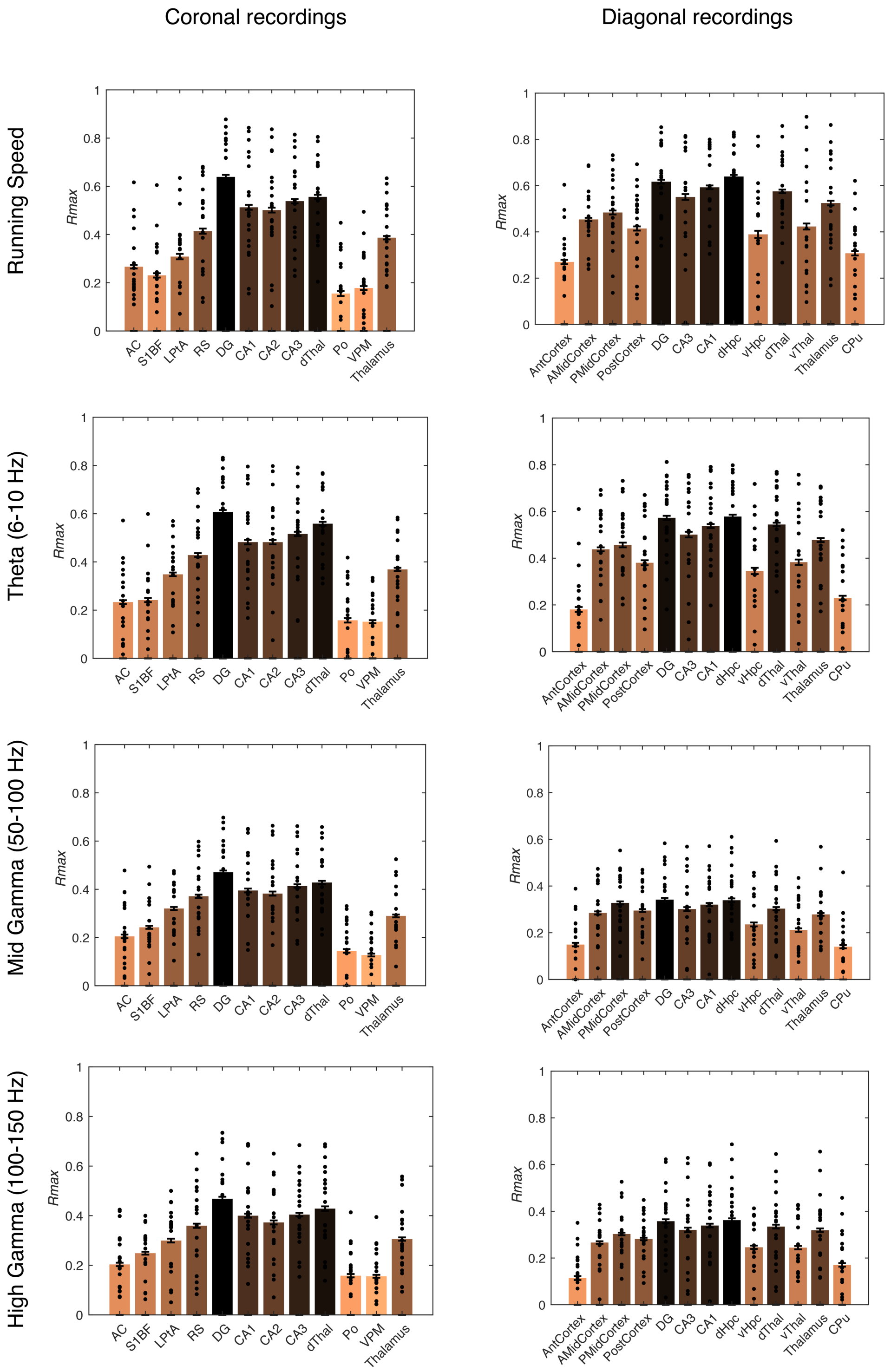

Supplementary Figure 4: Comparative analysis of seed-based correlations

In Figure 3, we computed speed-CBV cross-correlations and extracted R_{max} and T_{max} for all CBV regional response, which provides a measure of the proportion of variance in CBV fluctuations that is explained by speed. We also computed LFP-CBV cross-correlations for three different hippocampal LFP signals (from top to bottom): theta power (6-10 Hz), mid gamma power (50-100 Hz) and high gamma power (100-150 Hz), which have already been shown to correlate with CBV fluctuations. Here, we display the R_{max} distributions for 12 regions for all coronal recordings (left lane n=22 recordings, 7 animals) and for 13 regions for all diagonal recordings (right lane, n=20 recordings, 4 animals). LFP signals have been smoothed with a gaussian window of 0.5 s to extract envelope of the band-pass filtered LFP signal. Error bars correspond to the mean values +/- sem. Source data are provided as a Source Data file.

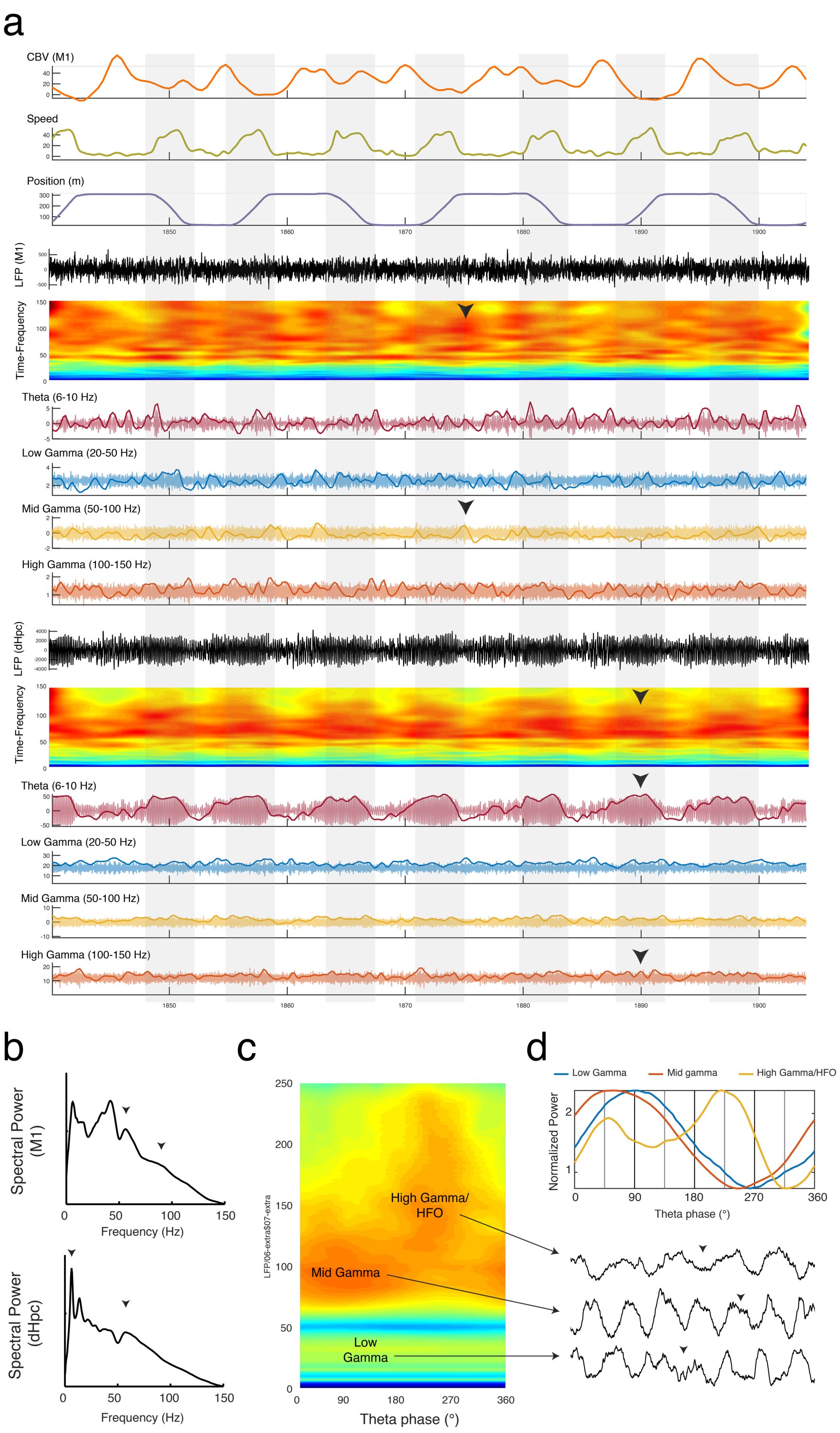

Supplementary Figure 5: Details of motor cortex and hippocampal LFP recording profiles during locomotion

(a) Time-frequency analysis of the hippocampal and motor cortex LFP during running for the selected hippocampal and motor cortex sites. (Top to bottom) CBV signal in the primary motor cortex (M1), Animal Speed and Position along the long axis (cm), Raw LFP, Time-frequency spectrogram, Bandpass-filtered LFP and power envelopes in the theta (6-10 Hz), low gamma (20-50 Hz), mid gamma (50-100 Hz), high gamma (100-150 Hz) bands for M1 (middle) and dorsal hippocampus (bottom) recording sites. Instantaneous power envelope is computed by temporal smoothing (gaussian kernel 1.0 s). Runs are displayed in shaded grey. Note the prominent inhibition in M1 CBV

signal during locomotion as well as bursts of theta (hippocampus) and fast gamma (hippocampus and motor cortex) oscillations concurrent with each individual run (black arrows).

(b) Power spectral density of the hippocampal and motor cortex LFP recording during locomotion. Note the two prominent peaks (arrows) in the theta and gamma bands for the hippocampal LFP and in the gamma band for the cortical one.

(c) Theta-phase time-frequency spectrogram of the hippocampal LFP. Theta-phase is extracted by linear interpolation of the zero-crossing function of theta-filtered LFP signal. Note the very clear distinction between the three different gamma sub-bands, in accordance with previous studies[50].

(d) Top: Phase-amplitude cross-frequency coupling between theta and gamma bands in the hippocampal LFP show three distinct sub-bands (0°: theta trough). Low gamma is maximal around at the ascending phase of theta [180°-270°]. Mid gamma is maximal at the peak [90°-180°] and high gamma is maximal close to the trough [300°-360°]. Bottom: Three typical illustrative traces of individual gamma events, all of which occur during locomotion.

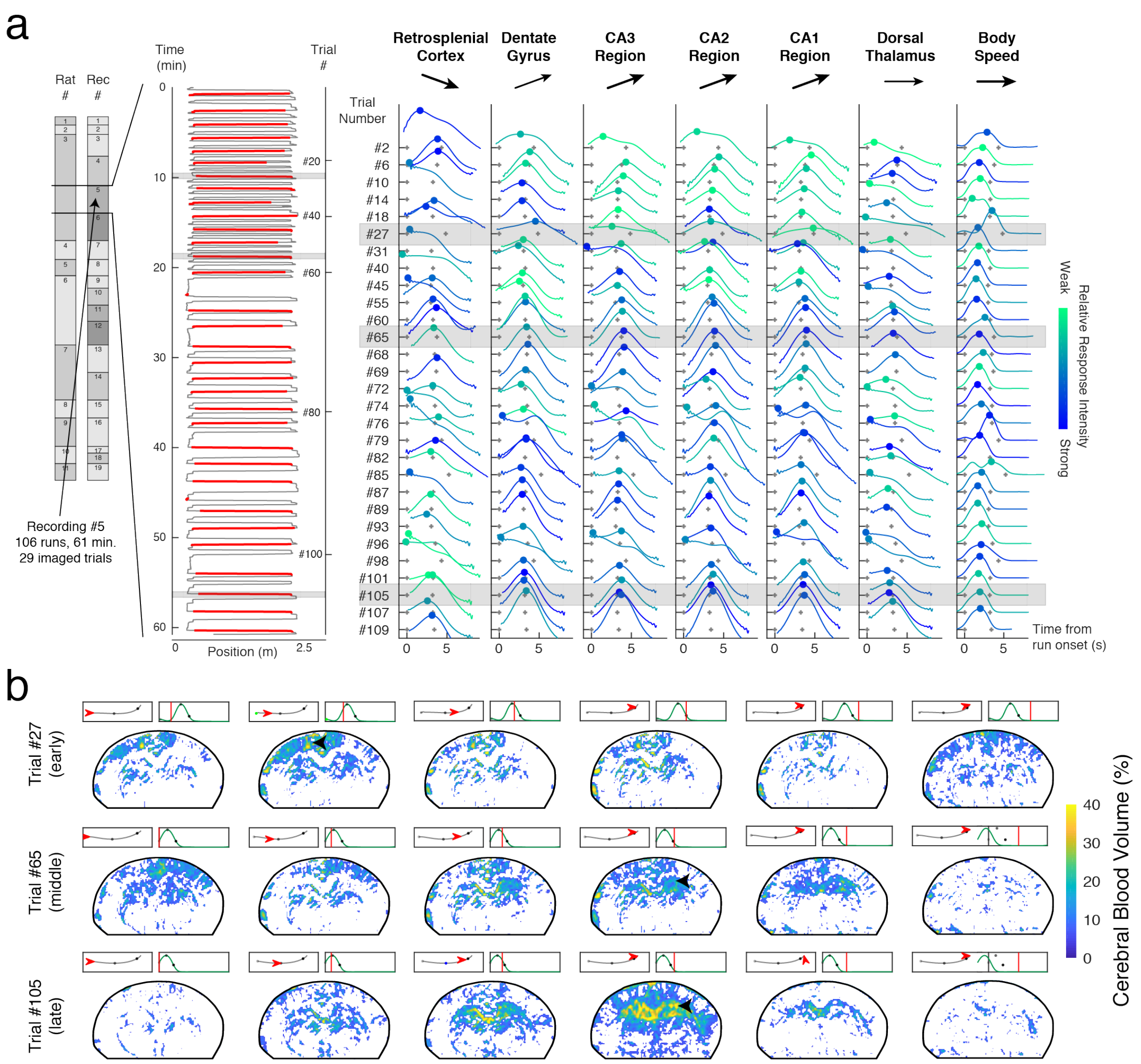

Supplementary Figure 6: Single-trial hemodynamic responses are strongly modulated over the course of a single session

(a) Inter-trial analysis of locomotion-related hemodynamics is shown for a single recording session, totalizing 106 runs, 29 of which have been captured by fUS. Left: Animal position on the long axis of the linear track versus time. Recorded runs are shown in red (Burst mode imposes a dead time between two 12 s insonification epochs). Right: Regional hemodynamic responses during all 29 imaged trials for the six strongly responsive regions shown in Fig. 2. (retrosplenial cortex, dentate gyrus, CA1, CA2, CA3 and dorsal thalamus) All six brain regions display inter-trial variability while running speed remained constant (right). For each column, trials are color-coded normalized to the weakest and strongest responses within the session (blue: strong, light green: weak response). Black arrows indicate the tendency for the vascular response to either potentiate, remain stable or decrease (bold: strong tendency). While body speed or dorsal thalamus hemodynamics displayed an even repartition of weak and strong responses along the whole session, hemodynamics in the retrosplenial cortex on the one hand and in the four sub-regions of the hippocampus on the other hand showed a strong bias: strong cortical activations occurred consistently in the early runs while strong hippocampal trials were gathered in the last 2/3 of the session. This effect was even stronger in the CA regions compared to dentate gyrus.
(b) Spatiotemporal dynamics of 3 trials shaded in (a), each taken during the early (trial 6), middle (trial 12) and last (trial 27) portion of the recording. In the early run, locomotion strongly activated various cortical regions including retrosplenial cortex together with dorsal thalamus and dentate gyrus. In the intermediate run, the cortical activation was weakened and the hippocampal activation spread to the downstream (CA1/CA3) regions of the hippocampus (black arrow). In the late run, cortical response was absent, thalamic activation was weakened, while the full dorsal hippocampus was recruited.

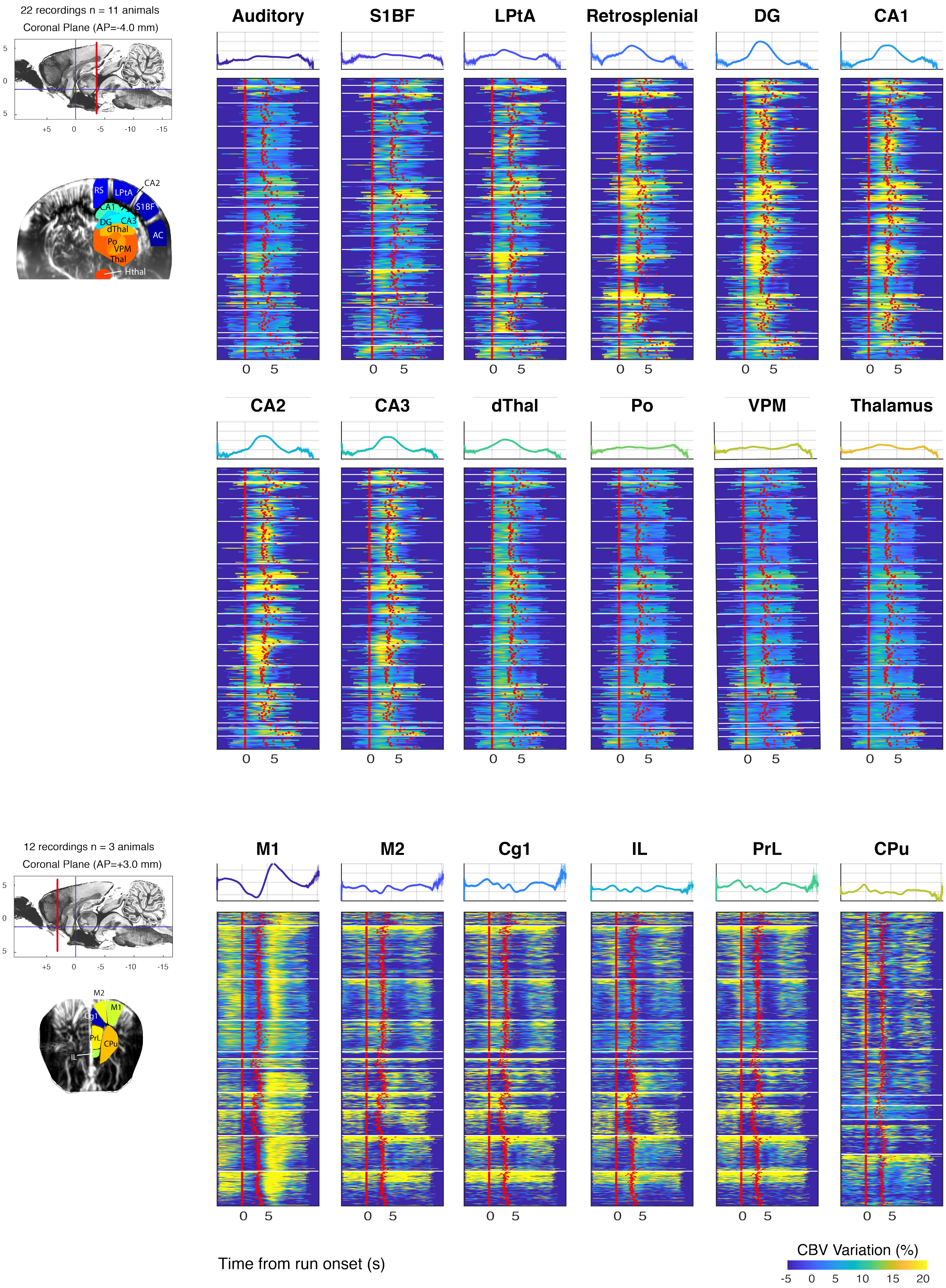

Supplementary Figure 7: Brain-wide hemodynamic modulation is directly observable in individual responses to single runs in hippocampal and cortical sites.

Bulk representation of single-trial hemodynamic responses to locomotion in all brain regions recorded on the two coronal planes (Top: Plane 1, Bregma = -4.0 mm – Bottom: Plane 2, Bregma = +3.0 mm) (RS: retrosplenial cortex, LPtA: lateral parietal associative cortex, S1BF: primary somatosensory

barrel field, AC: auditory cortex, DG: dentate gyrus, CA1-CA2-CA3 region, dThal: dorsal Thalamus, Po: Posterior Thalamic Nucleus, VPM: Ventroposterior Thalamic Nucleus, Thal: Thalamus, Hthal: Hypothalamus, M1: primary motor cortex, M2: secondary motor cortex, Cg1: anterior cingulate cortex, PrL: prelimbic cortex, IL: infralimbic cortex, CPu: caudate putamen). A total of 384 trials (19 recordings, 7 animals) for Plane 1 and 928 trials (15 recordings, 3 animals) for Plane 2 was acquired across many days. For each run, the onset of movement is used as a temporal reference (zero-timing) and all trials are aligned to run onset (see Methods). Strongest activations are found in all subfields of the dorsal hippocampus and retrosplenial and lateral parietal cortices. Recording sessions are separated by horizontal white lines and grouped by individuals. Note the very clear and strong inter-trial variability within the same recording session in hippocampal (hemodynamic potentiation) and cortical (hemodynamic depression) regions, with strongest hippocampal activations in the late trials of each recording while cortical activations are stronger in the early trials. Source data are provided as a Source Data file. Reprinted from reference 88, Copyright (2014), with permission from Elsevier.

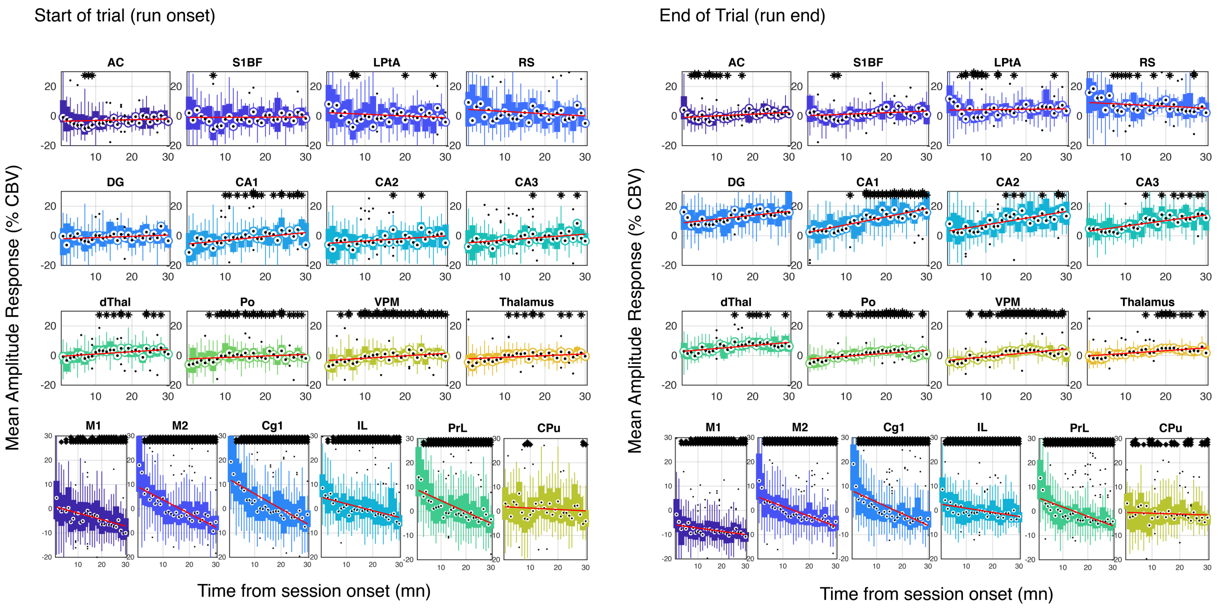

Supplementary Figure 8: Hemodynamic modulation affects differently the early and late components of single-trial hemodynamic responses.

For each trial, we extract either the median in a 200-ms window centered on trial start (left) or trial end (right). Each trial is then grouped in 1mn bin according to their timing from session set (0 refers to the first run in each recording) for 18 regions across all individuals (n=10 animals, 37 recordings total). Left columns represent early runs while right columns represent late runs. Note that the hemodynamic modulation affects the early part of the CBV response (trial start) less than the late part of the CBV response (trial end), in the dorsal thalamus and in the dorsal hippocampus. Conversely, the early part of the CBV response in more strongly modulated than the late part in frontal cortical regions. (Top: Plane 1, Bregma = -4.0 mm – Bottom: Plane 2, Bregma = +3.0 mm) (RS: retrosplenial cortex, LPtA: lateral parietal associative cortex, S1BF: primary somatosensory barrel field, AC: auditory cortex, DG: dentate gyrus, CA1-CA2-CA3 region, dThal: dorsal Thalamus, Po: Posterior Thalamic Nucleus, VPM: Ventroposterior Thalamic Nucleus, Thal: Thalamus, Hthal: Hypothalamus, M1: primary motor cortex, M2: secondary motor cortex, Cg1: anterior cingulate cortex, PrL: prelimbic cortex, IL: infralimbic cortex, CPu: caudate putamen). Source data are provided as a Source Data file.

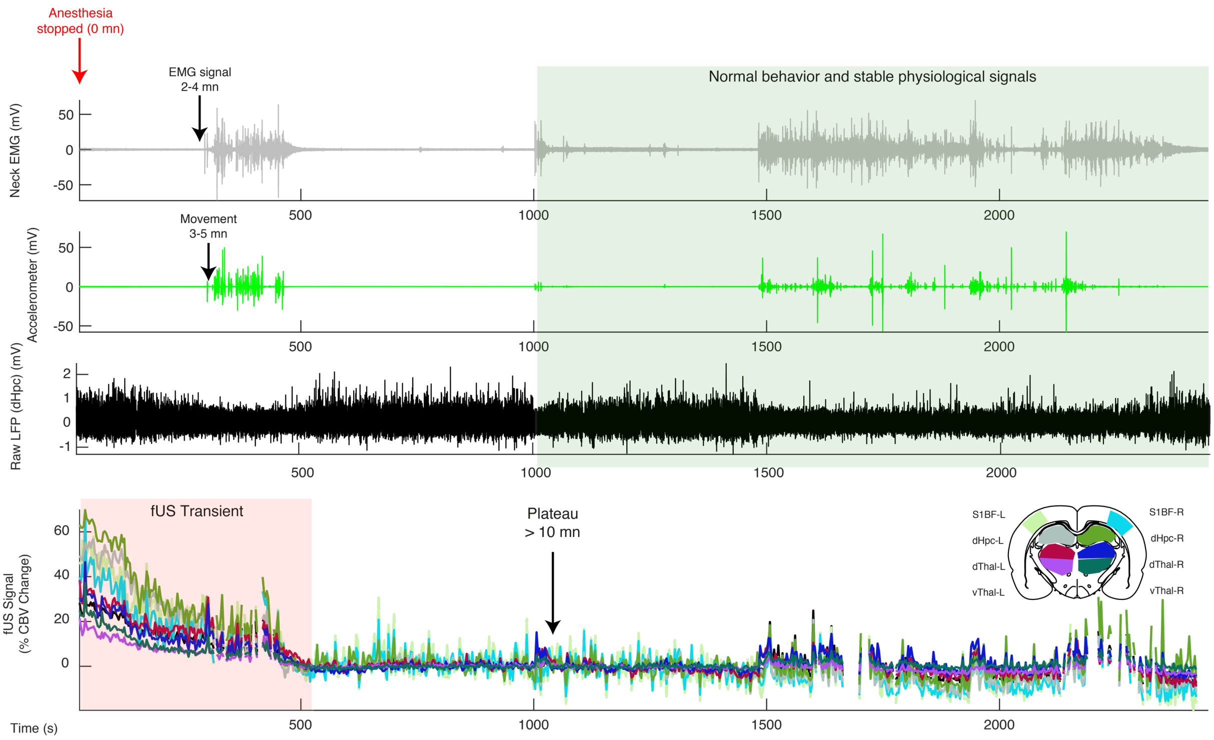

Supplementary Figure 9: Vascular dynamics during recovery from isoflurane anesthesia

Typical EEG-fUS-VIDEO recording during recovery from anesthesia. The animal typically wakes up a few minutes after isoflurane was stopped, with EMG signal preceding movement onset. The vascular dynamics show a transient exponential decay (shaded red box) that lasts for another 5 to 10 minutes before reaching a plateau, approximately 10 min after anesthesia was stopped. We waited for another 30 min (40 min total) and made sure behavior and electrophysiological signals were stable and normal (shaded green box) before starting running sessions to clear any residual effect from anesthesia.
