# Supplementary material for: Adaptive modulation of brain hemodynamics across stereotyped running episodes
Source: Nat Commun. 2020 Dec 3;11:6193. doi: 10.1038/s41467-020-19948-7 (PMC7713412; doi:10.1038/s41467-020-19948-7)
Supplement: Supplementary file 3 — Description of Additional Supplementary Files [file 41467_2020_19948_MOESM3_ESM.pdf]

<u>List of Supplementary Movies</u>

Supplementary Movie 1: Dynamic activation sequence associated with locomotion in posterior brain regions at 10 milliseconds resolution (Coronal plane – Bregma -4.0mm)

Supplementary Movie 2: Dynamic activation sequence associated with locomotion across the full hippocampus at 10 milliseconds resolution (Diagonal plane – Delta = 45°)

Supplementary Movie 3: Dynamic inhibition/activation sequence associated with locomotion in the primary motor cortex (Coronal plane – AP = +3.0mm)

Supplementary Movie 4: Early-Mid-Late single runs acquired during locomotion in posterior brain regions (Coronal plane – Bregma -4.0mm – Rat 1)

Supplementary Movie 5: Early-Mid-Late single runs acquired during locomotion in posterior brain regions (Coronal plane – Bregma -4.0mm – Rat 2)

Supplementary Movie 6: Early-Mid-Late single runs acquired during locomotion in anterior brain regions (Coronal plane – Bregma +3.0mm – Rat 3)
