# Supplementary material for: Adaptive modulation of brain hemodynamics across stereotyped running episodes
Source: Nat Commun. 2020 Dec 3;11:6193. doi: 10.1038/s41467-020-19948-7 (PMC7713412; doi:10.1038/s41467-020-19948-7)
Supplement: Supplementary file 4 — Supplementary Movie 1 [file 41467_2020_19948_MOESM4_ESM.pdf]

nature research

Corresponding author(s): BERGEL

Last updated by author(s): Oct 20, 2020

Reporting Summary

Nature Research wishes to improve the reproducibility of the work that we publish. This form provides structure for consistency and transparency in reporting. For further information on Nature Research policies, seeAuthors & Referees and theEditorial Policy Checklist .

Statistics

For all statistical analyses, confirm that the following items are present in the figure legend, table legend, main text, or Methods section.

n/a	Confirmed	
☐	☒	The exact sample size (*n*) for each experimental group/condition, given as a discrete number and unit of measurement
☐	☒	A statement on whether measurements were taken from distinct samples or whether the same sample was measured repeatedly
☐	☒	The statistical test(s) used AND whether they are one- or two-sided<br>*Only common tests should be described solely by name; describe more complex techniques in the Methods section.*
☒	☐	A description of all covariates tested
☐	☒	A description of any assumptions or corrections, such as tests of normality and adjustment for multiple comparisons
☐	☒	A full description of the statistical parameters including central tendency (e.g. means) or other basic estimates (e.g. regression coefficient) AND variation (e.g. standard deviation) or associated estimates of uncertainty (e.g. confidence intervals)
☐	☒	For null hypothesis testing, the test statistic (e.g. *F*, *t*, *r*) with confidence intervals, effect sizes, degrees of freedom and *P* value noted<br>*Give P values as exact values whenever suitable.*
☒	☐	For Bayesian analysis, information on the choice of priors and Markov chain Monte Carlo settings
☒	☐	For hierarchical and complex designs, identification of the appropriate level for tests and full reporting of outcomes
☐	☒	Estimates of effect sizes (e.g. Cohen's *d*, Pearson's *r*), indicating how they were calculated

Our web collection on statistics for biologists contains articles on many of the points above.

Software and code

Policy information about availability of computer code

Data collection	fUS data was collected via custom code.<br>For the 1st set of recordings, LFP and video were collected using custom code developed in Labview 2016 (National Instruments, Austin, TX, USA). For the 2nd set of recordings, LFP and video were collected via the Cereplex Direct Software Suite (version 7.0.6.0, Blackrock Mircrosystems, Salt Lake City, UT, USA). All non-commercial custom source codes used in this study are protected by INSERM and can only be shared upon request, with the written agreement of INSERM.
Data analysis	All analysis were performed using custom code under MATLAB (version R2017b). NPMK package (version 4.5.3.0) was used to import the raw LFP Data into MATLAB. Simplex method was implemented via custom code in LabView 2016. All custom codes used for the analysis of functional ultrasound/EEG/video data used in this study are protected by INSERM and can only be shared upon request, with the written agreement of INSERM.

For manuscripts utilizing custom algorithms or software that are central to the research but not yet described in published literature, software must be made available to editors/reviewers. We strongly encourage code deposition in a community repository (e.g. GitHub). See the Nature Research guidelines for submitting code & software for further information.

Data

Policy information about availability of data

All manuscripts must include a data availability statement. This statement should provide the following information, where applicable:

- Accession codes, unique identifiers, or web links for publicly available datasets
- A list of figures that have associated raw data
- A description of any restrictions on data availability

All data and software supporting the findings of this study are available from the corresponding authors upon reasonable request.

Field-specific reporting

Please select the one below that is the best fit for your research. If you are not sure, read the appropriate sections before making your selection.

[x] Life sciences [ ] Behavioural & social sciences [ ] Ecological, evolutionary & environmental sciences

For a reference copy of the document with all sections, see nature.com/documents/nr-reporting-summary-flat.pdf

Life sciences study design

All studies must disclose on these points even when the disclosure is negative.

Sample size	No preliminary sample size calculation was performed in this study. The sample size was taken as high as possible considering the experimental constraints (difficult surgical procedure, precise electrode implantation, habituation and training required for the locomotion task). Minimum sample size was set to 3 animals and to 15 recordings for each group, to ensure proper statistical analysis.
Data exclusions	No animals were excluded from the analysis. For Figures 6, 7 only recordings (15/25) with a sufficient number of trials (10) were kept in the analysis because it was not possible compute Pearson correlation coefficients if the number of trials was too low.
Replication	No replication was performed in this study due to difficult experimental constraints named above.
Randomization	No randomization was necessary. Each animal was used as its own control in each recording group.
Blinding	No blinding was necessary. Each animal was used as its own control in each recording group.

Reporting for specific materials, systems and methods

We require information from authors about some types of materials, experimental systems and methods used in many studies. Here, indicate whether each material, system or method listed is relevant to your study. If you are not sure if a list item applies to your research, read the appropriate section before selecting a response.

Materials & experimental systems

n/a	Involved in the study	
[x]	[ ]	Antibodies
[x]	[ ]	Eukaryotic cell lines
[x]	[ ]	Palaeontology
[ ]	[x]	Animals and other organisms
[x]	[ ]	Human research participants
[x]	[ ]	Clinical data

Methods

n/a	Involved in the study	
[x]	[ ]	ChIP-seq
[x]	[ ]	Flow cytometry
[x]	[ ]	MRI-based neuroimaging

Animals and other organisms

Policy information about studies involving animals; ARRIVE guidelines recommended for reporting animal research

Laboratory animals	Sprague Dawley Rats, Male, 10-14 weeks old
Wild animals	No wild animals were used in this study.
Field-collected samples	No field-collected samples were used in this study.
Ethics oversight	All animals received humane care in compliance with the European Communities Council Directive of 2010 (2010/63/EU). The experimental protocol used in this study was extensively reviewed an approved by the French 'Comité d'Ethique pour l'Experimentation Animale' n°59 Paris Centre et Sud, under the reference number 2018061320381023.

Note that full information on the approval of the study protocol must also be provided in the manuscript.
